# Rational Construction of Uniform CoNi-Based Core-Shell Microspheres with Tunable Electromagnetic Wave Absorption Properties

**DOI:** 10.1038/s41598-018-21047-z

**Published:** 2018-02-16

**Authors:** Na Chen, Jian-Tang Jiang, Cheng-Yan Xu, Shao-Jiu Yan, Liang Zhen

**Affiliations:** 1 School of Materials Science and Engineering, Harbin Institute of Technology, Harbin, 150001 China; 2 MOE Key Laboratory of Micro-System and Micro-Structures Manufacturing, Harbin Institute of Technology, Harbin, 150080 China; 3 Beijing Institute of Aeronautical Materials, Beijing, 100095 China

## Abstract

Core-shell particles with integration of ferromagnetic core and dielectric shell are attracting extensive attention for promising microwave absorption applications. In this work, CoNi microspheres with conical bulges were synthesized by a simple and scalable liquid-phase reduction method. Subsequent coating of dielectric materials was conducted to acquire core-shell structured CoNi@TiO_2_ composite particles, in which the thickness of TiO_2_ is about 40 nm. The coating of TiO_2_ enables the absorption band of CoNi to effectively shift from K_u_ to S band, and endows CoNi@TiO_2_ microspheres with outstanding electromagnetic wave absorption performance along with a maximum reflection loss of 76.6 dB at 3.3 GHz, much better than that of bare CoNi microspheres (54.4 dB at 17.8 GHz). The enhanced EMA performance is attributed to the unique core-shell structures, which can induce dipole polarization and interfacial polarization, and tune the dielectric properties to achieve good impedance matching. Impressively, TiO_2_ coating endows the composites with better microwave absorption capability than CoNi@SiO_2_ microspheres. Compared with SiO_2_, TiO_2_ dielectric shells could protect CoNi microspheres from merger and agglomeration during annealed. These results indicate that CoNi@TiO_2_ core-shell microspheres can serve as high-performance absorbers for electromagnetic wave absorbing application.

## Introduction

Electromagnetic wave absorbing (EMA) materials have attracted much attention in past few decades because of the increasing requirement for conquering the electromagnetic interference and electromagnetic disclosure^1–5^. EMA materials with wide absorption bands, strong reflection loss and satisfactory weather resistance are urgently required^6–9^. A variety of materials have been explored for using as EMA fillers. Ferromagnetic metal/alloy particles, such as CoNi particles, attract considerable interest as microwave absorbers due to their unique ferromagnetic features, including high saturation magnetization, high Snoek’s limit, and high magnetocrystalline anisotropy^10,11^. For CoNi particles, the EMA performance is strongly influenced by the particle morphology and microstructure^12^. Great efforts have been devoted to design and synthesize CoNi particles with various shapes, such as nanoparticles^13^, microspheres^14^, chains^15,16^, wires^17,18^, flowers^19–21^, nanotubes^22,23^ and nanoleaves^24^. These studies preliminarily investigated the effects of morphology on EMA properties via the application of CoNi particles, and confirmed that size and shape of particles had an obvious effect on EMA performance. Specially, according to the electromagnetic wave propagation theory, the modified surface could effectively tailor the EMA performance through surface scattering effects^12^. However, systematic investigation focusing on the impacts of configuration and surface morphology on the electromagnetic properties is scarce. Additionally, CoNi materials usually suffer from ease of oxidation, resulting in limited practical applications.

To fabricate core-shell structured composite particles that integrate the ferromagnetic components and dielectric components together at sub-micro/nano scales is believed to be a promising approach to solve the above problems^25,26^. The protecting shells on particles’ surface yield multiple interfaces and isolates particles from contacting each other, which contributes to the dielectric dissipation^27^, suppresses eddy current^28^ and also avoids decay-induced performance degradation. Improved EM properties, either in amplitude or in spectrum characteristics, were observed in this catalog of composite materials, suggesting the great potential of core-shell composite structures^29–31^. For example, Zhang *et al*. synthesized core-shell Ni-TiO_2_ composite microspheres with enhanced microwave absorption properties, which arises from multiple interfacial polarization and high thermal conductivity of rutile TiO_2_^32^. Ren *et al*. fabricated three-dimensional SiO_2_@Fe_3_O_4_ core-shell nanorods array/graphene architecture. The significantly improved dielectric loss of SiO_2_@Fe_3_O_4_ composite is attributed to the dipolar polarization and interfacial polarization^33^. Li *et al*. successfully prepared FeCo/graphene hybrids with remarkable improvement in permeability and permittivity, which leads to remarkable enhancement in EM absorption properties^34^.

Among numerous dielectrics shell materials, including carbon materials, SnO_2_, BaTiO_3_, TiO_2_, SiO_2_ as well as polymers, TiO_2_ as an important semiconductor material^35–37^ has been widely explored for electromagnetic wave absorption applications due to its dominant dipolar polarization and corresponding relaxation phenomena, which contributes to the dielectric loss mechanism^38,39^. Meanwhile, TiO_2_ is also attractive as a coating material to enhance the microwave absorption performance since it owns high dielectric constant^40^. Accordingly, it is expected that the interface between the magnetic core and TiO_2_ shell could produce some intriguing interactions, which could extremely enhance EMA properties of ferromagnetic particles.

The purpose of this work was to design and fabricate core-shell composites to achieve materials with outstanding EMA performance. A facile and efficient method was developed to prepare composite microspheres with CoNi as cores and TiO_2_ as shells, in which CoNi cores can contribute to the magnetic loss, while TiO_2_ shells can contribute to the dielectric loss. The microwave absorption properties of CoNi microspheres and core-shell composites microspheres were evaluated. The results suggest that CoNi@TiO_2_ microspheres possess outstanding microwave absorption capabilities. Our findings give insights into the understanding of the effects of core-shell structure on the microwave absorption performance, which can be extended to other ferromagnetic metals and ferrites for EMA applications.

## Results and Discussion

**Figure 1:**
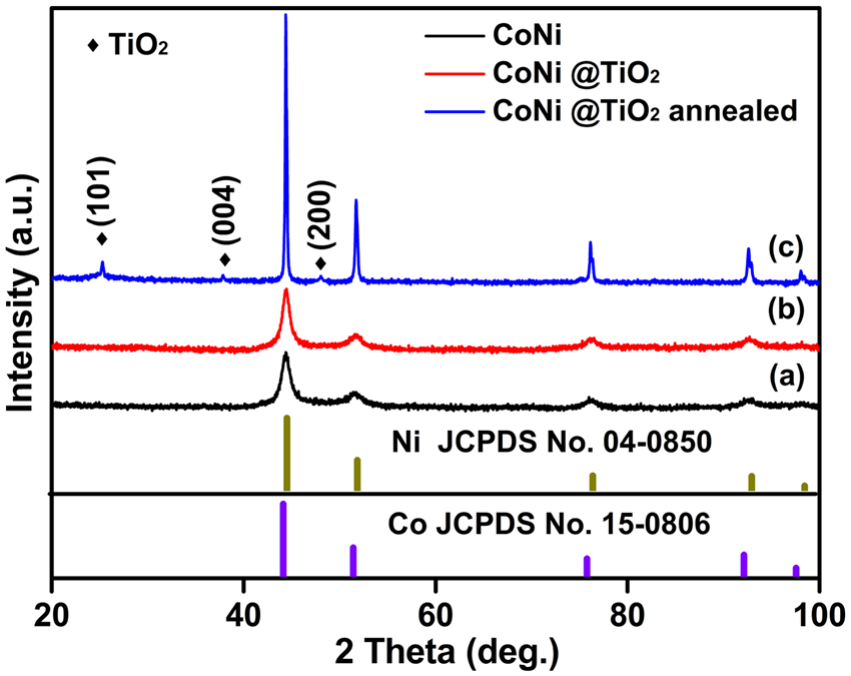
XRD patterns of (**a**) as-prepared CoNi microspheres, (**b**) CoNi@TiO_2_ core-shell microspheres and (**c**) CoNi@TiO_2_ core-shell microspheres annealed at 600 °C.

The crystal structure of as-prepared CoNi microspheres and core-shell structure composites were characterized by XRD. As shown in Fig. 1a, four strong peaks (2θ = 44.4°, 51.6°, 76.3° and 92.7°) are observed in the XRD pattern, which can be indexed to the (111), (200), (220) and (311) planes of face-centered cubic (*fcc*) phase CoNi^41^, respectively (JCPDS no. 15–0806 for *fcc* Co, JCPDS no. 04–0850 for *fcc* Ni). No other characteristic peaks are observed in the pattern, indicating the high purity of as-obtained CoNi microspheres. The characteristics peaks of TiO_2_ cannot be detected in the XRD pattern of as-synthesized CoNi@TiO_2_ (Fig. 1b), suggesting that the TiO_2_ shells should be amorphous. After annealed at 600 °C for 2 h, three characteristic diffraction peaks were found to be located at 2θ of 25.3°, 37.8° and 48.0°, corresponding to the (101), (004) and (200) crystal planes of anatase TiO_2_ (JCPDS No. 21–1272), as shown in Fig. 1c. Meanwhile, XRD peaks of annealed microspheres are much sharper and stronger, demonstrating the improvement of crystallinity for CoNi@TiO_2_ microspheres. The crystal structure of CoNi@SiO_2_ microspheres were also characterized by XRD (Fig. S1). No characteristic peaks corresponding to crystalline SiO_2_ can be detected in the XRD patterns, indicating that SiO_2_ shells should be amorphous states.

**Figure 2:**
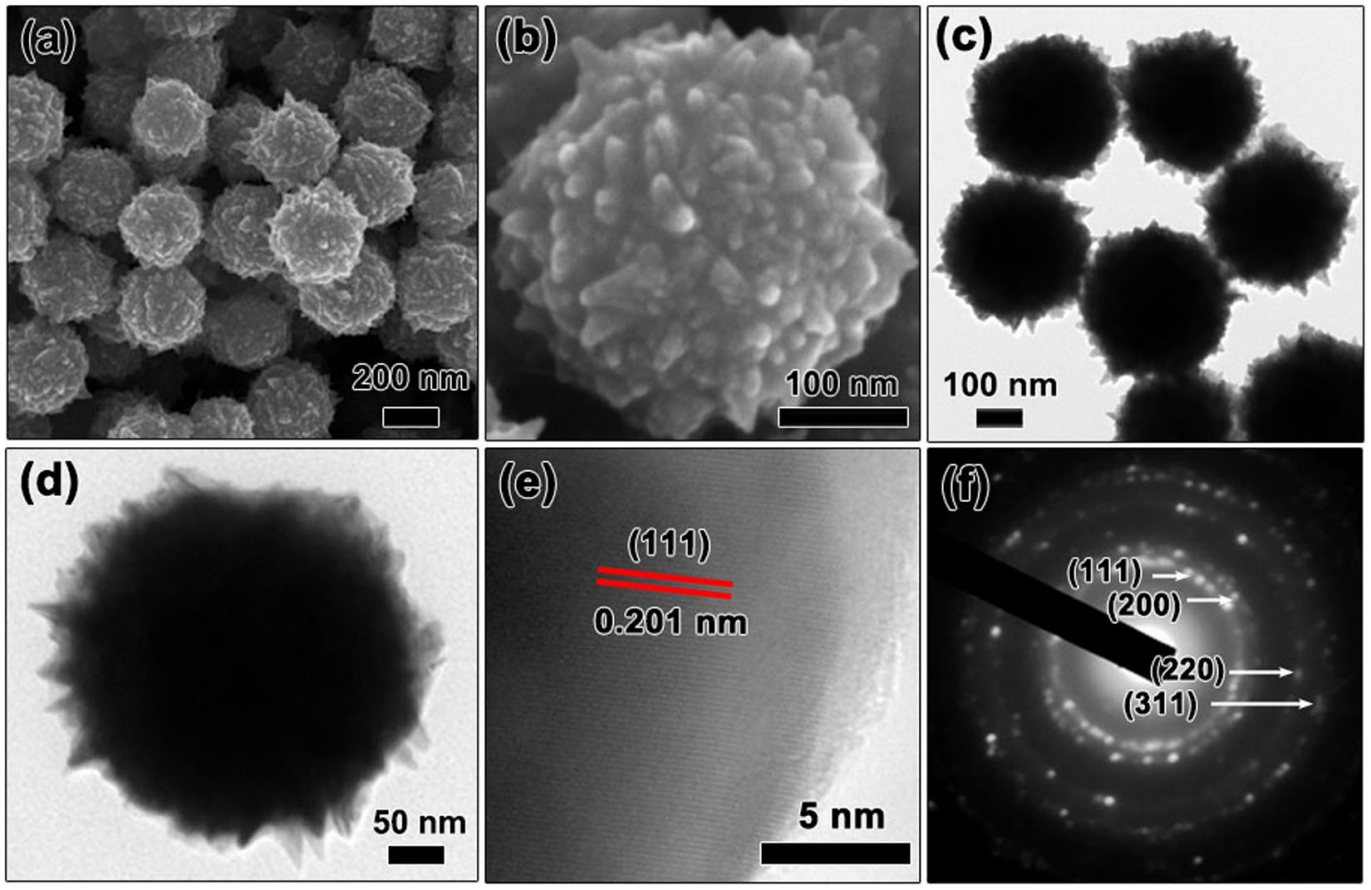
Characterization of as-synthesized CoNi microspheres. (**a**,**b**) SEM images; (**c**,**d**) TEM images; (**e**) HRTEM image; (**f**) SAED pattern.

The morphology of CoNi microspheres was observed by SEM and TEM. SEM image in Fig. 2a and TEM image in Fig. 2c reveal that the as-prepared CoNi particles are uniform microspheres with an average diameter of about 300 nm. Interestingly, it can be observed that conical bulges with a length of 5–15 nm emerge on the pristine CoNi microspheres, as shown in Fig. 2b and d. Energy dispersive X-ray spectroscopy (EDS) analysis was performed to check the compositions (Fig. S2). The atomic ratio of Co/Ni (50.2:49.8) is approximately 1:1, very close to the stoichiometry of CoNi. Element mappings obtained from EDS analysis also suggest that the distribution of Co and Ni elements is rather homogeneous in entire microsphere. High-resolution TEM (HRTEM) image taken from a single microsphere reveals the well-resolved lattice fringes corresponding to the (111) plane (*d* = 0.201 nm) of cubic CoNi, as described in Fig. 2e. Selected-area electron diffraction (SAED) pattern depicted in Fig. 2f shows distinct diffraction rings corresponding to (111), (200), (220) and (311) crystallographic planes of cubic CoNi, in accordance with XRD analysis. HRTEM and SAED results clearly prove the highly crystalline of CoNi microspheres. Based on SEM and TEM analysis, it is confirmed that CoNi microspheres with conical bulges surface have been successfully fabricated via liquid phase reduction method. The unique and novel conical bulge of CoNi microspheres is expected to enhance EMA performance.

**Figure 3:**
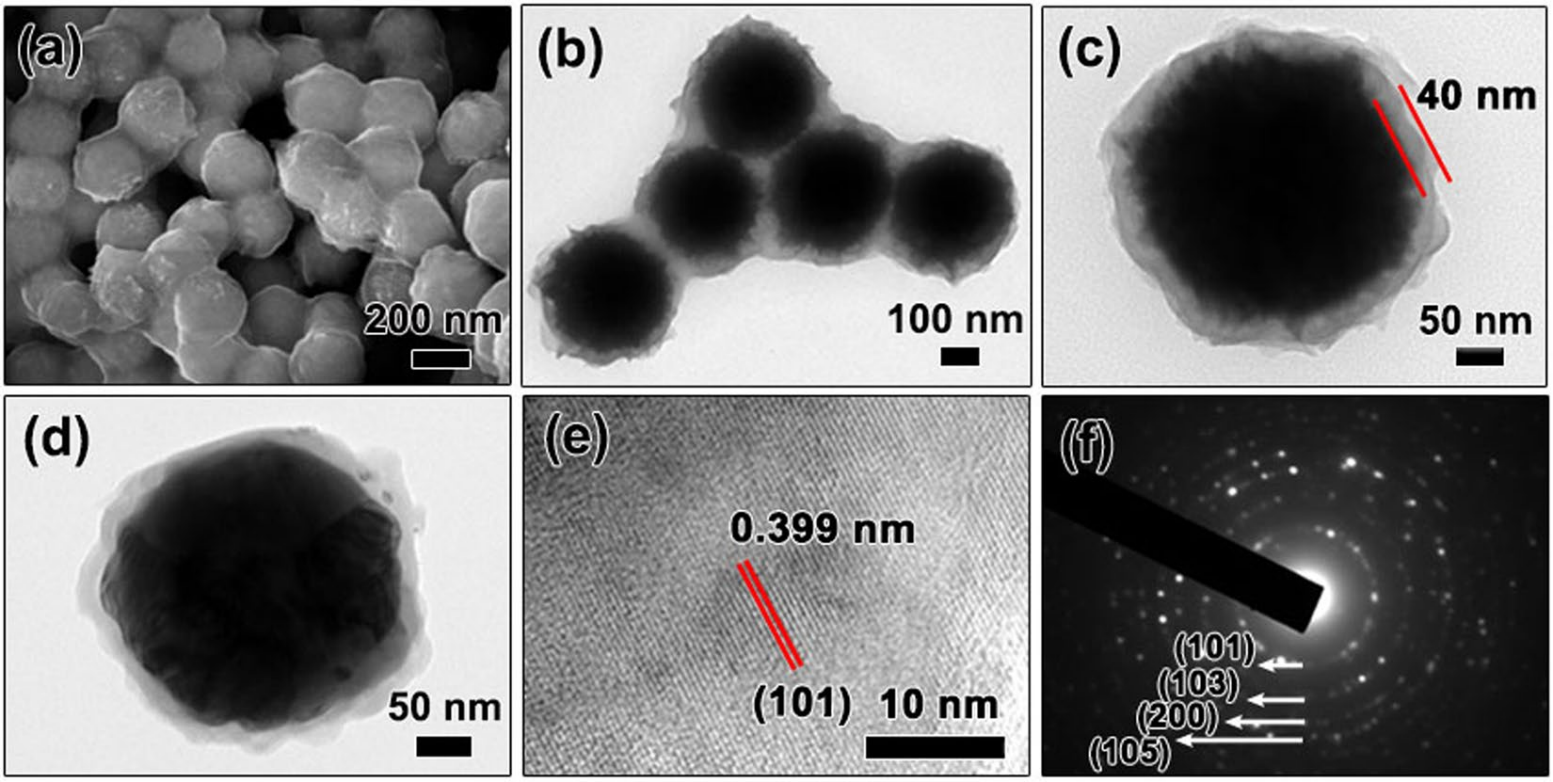
Characterization of the structure and morphology of CoNi@TiO_2_ core-shell microspheres. (**a**) SEM and (**b**) TEM image of as-prepared CoNi@TiO_2_ microspheres. (**c**) TEM image of a single CoNi@TiO_2_ microsphere. (**d**) TEM image of CoNi@TiO_2_ microsphere annealed at 600 °C. (**e**) HRTEM image and (**f**) SAED pattern of TiO_2_ shell after annealed.

CoNi microspheres are coated by TiO_2_ shells through a sol-gel method. The microstructure and morphology of CoNi@TiO_2_ composites microspheres were characterized by SEM and TEM. SEM and TEM images in Fig. 3a,b show the uniform size distribution and core-shell structure of CoNi@TiO_2_ composites particles. The microsphere morphology characteristics of CoNi could be well maintained after TiO_2_ coating. It is worth noting that some conglomerates containing a few CoNi@TiO_2_ microspheres are observed, as shown in Fig. 3a. CoNi microspheres are supposed to aggregate together when wrapped in TiO_2_ during the sol-gel process, leading to the slender shape and close-packed microstructure of these conglomerates (Fig. 3b). The intervals between CoNi particles are 10–50 nm, which enable the local conducting within the conglomerates. The EDS spectrum as well as elemental mappings obtained from an individual CoNi@TiO_2_ microsphere in Fig. S3 confirms homogeneous distribution of Co, Ni, O and Ti elements. From the high-magnification SEM image in Fig. S3b, it could be clearly seen that the surface of CoNi@TiO_2_ is different from that of CoNi microsphere. The core-shell microspheres have a nearly flat surface, indicating that TiO_2_ shell covers the surface of CoNi particles. TEM image in Fig. 3c verifies the typical core-shell structure of CoNi@TiO_2_, in which an outer layer of about 40 nm in thickness can be clearly distinguished. SEM image in Fig. S4 shows that the morphology of CoNi@TiO_2_ microspheres was well retained after annealed at 600 °C. The sizes of CoNi core exhibit negligible change and the thickness of TiO_2_ shell remains to be about 40 nm (Fig. 3d). The corresponding HRTEM image taken from TiO_2_ shell of a single annealed microsphere is exhibited in Fig. 3e. The lattice fringe with distance of 0.399 nm is in good accordance with the (101) plane of anatase TiO_2_. SAED pattern of TiO_2_ shell in Fig. 3f confirms that TiO_2_ is typical anatase phase with diffraction rings corresponding to the (101), (103), (200) and (105) planes, respectively. These results suggest that coating of CoNi microspheres with TiO_2_ shells could be successfully carried out by using a sol-gel method, and the annealing at high temperature (see XRD pattern in Fig. S5 and associated discussion in Supporting Information) can effectively tailor the crystal structure of TiO_2_ layers. More importantly, TiO_2_ shells can effectively protect and isolate CoNi microspheres from merger and aggregation in high-temperature annealing process.

SiO_2_ is also extensively used as a coating material since it is a good insulator. Our previous study indicated that the EMA properties of Co_7_Fe_3_ nanospheres could be improved by introduced SiO_2_ shells^42^. The morphology of as-obtained CoNi@SiO_2_ microspheres is rather uniform, as shown in Fig. S6. Elemental mappings obtained from EDS analysis (Fig. S7) reveal the homogeneous distribution of Co, Ni, O and Si elements. A close observation in Fig. S6c presents that the outcrop of conical bulges become blunt, revealing that SiO_2_ was successfully deposited onto CoNi surfaces. TEM image in Fig. S6d confirms that the SiO_2_ shell on the surface of CoNi microspheres is about 30 nm in thickness. The spherical morphology of CoNi@SiO_2_ was retained after annealed at 600 °C, however, the microspheres tend to merge and agglomeration is observed at local regions, as shown in Fig. S6e and f. The merging and resulted agglomeration of CoNi@SiO_2_ microspheres during annealing may cause the decrease of dielectric properties. These results suggest the TiO_2_ coating could protect CoNi microspheres from merger and agglomeration during annealed process more effectively compared with SiO_2_ coating. In the process of annealing, the difference of microstructure evolution between CoNi@TiO_2_ and CoNi@SiO_2_ should have a different effect on EMA performance. On the basis of above SEM and TEM analysis, it is confirmed that core-shell structure CoNi@TiO_2_ composites microspheres with TiO_2_ shells can be obtained through sol-gel process. It could be deduced that this unique core-shell structure is helpful to improve the EMA performance, which will be discussed in the following part.

**Figure 4:**
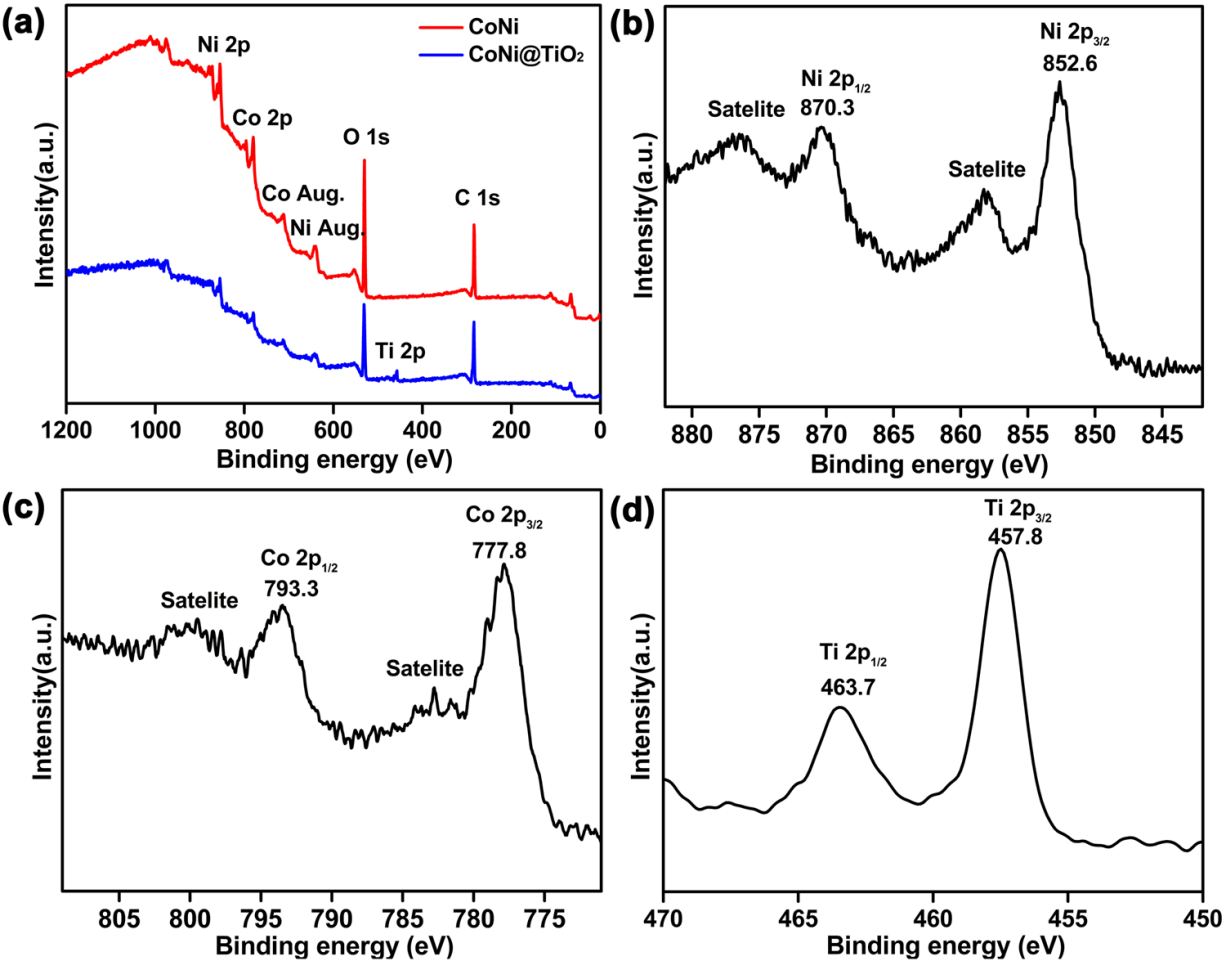
(**a**) XPS survey spectra of CoNi and CoNi@TiO_2_ microspheres. High-resolution XPS spectra of (**b**) Ni 2p and (**c**) Co 2p in as-prepared CoNi microspheres. (**d**) High-resolution XPS spectrum of Ti 2p in CoNi@TiO_2_ microspheres.

The surface compositions and element valence of CoNi and CoNi@TiO_2_ microspheres were investigated by XPS, and the results were shown in Fig. 4. The survey spectrum of CoNi microspheres in Fig. 4a reveals that the existence of Co, Ni, O and C elements. To further investigate the chemical states of Co and Ni elements, high resolution XPS spectra were conducted. Fig. 4b shows the high-resolution XPS spectrum of Ni 2p region. The peaks at 852.6 and 870.3 eV can be assigned to Ni 2p_3/2_ and Ni 2p_1/2_, suggesting the zero valent Ni^11^. The satellite peaks in the spectrum indicated the surface oxidation of nickel. Co 2p XPS spectrum in Fig. 4c shows two primary peaks at 777.8 eV (Co 2p_3/2_) and 793.3 eV (Co 2p_1/2_) corresponding to metallic cobalt^43^, along with satellite peaks at the higher binding energy region. These features belong to the characteristics of Co^2+^, implying the partial oxidation of cobalt. The presence of oxides cannot be detected by XRD measurement, suggesting their quite low percentage composition.

The survey spectrum of CoNi@TiO_2_ in Fig. 4a depicts the existence of Co, Ni, O, C and Ti elements, in agreement with the EDS results. High-resolution XPS spectrum of Ti 2p is shown in Fig. 4d. The peaks at 457.8 eV and 463.7 eV are assigned to Ti 2p_3/2_ and Ti 2p_1/2_, revealing the formation of TiO_2_ on the surface. The survey spectrum of CoNi@SiO_2_ in Fig. S8a demonstrates the presence of Co, Ni, O, C and Si elements. The peak at 103.5 eV in Fig. S8b is ascribed to Si 2p, indicating the formation of SiO_2_ on the surface. Based on the results of XPS, it can be concluded that CoNi microspheres were achieved and a thin surface layer were oxidized. TiO_2_ could be successfully coated on the surfaces of CoNi microspheres to form core-shell structure composite microspheres.

**Figure 5:**
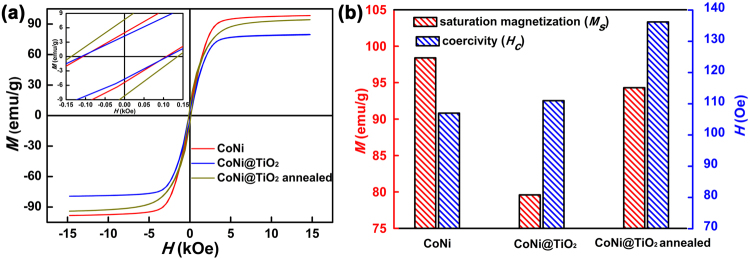
(**a**) Hysteresis loops of CoNi, CoNi@TiO_2_ and annealed CoNi@TiO_2_ microspheres measured at room temperature. The inset is an enlarged view of the hysteresis loops. (**b**) Magnetic properties of CoNi, CoNi@TiO_2_ and annealed CoNi@TiO_2_ microspheres.

The magnetic properties of CoNi and CoNi@TiO_2_ microspheres were measured on a VSM at room temperature, and the results are shown in Fig. 5. The saturation magnetization (*M*_*s*_), and coercivity (*H*_*c*_) of CoNi microspheres, CoNi@TiO_2_ and CoNi@TiO_2_ annealed are compared in Fig. 5b. The saturation magnetization of CoNi microspheres is 98.4 emu/g, about 12.1% lower than that of bulk CoNi (112 emu/g)^24^, which may be attributed to the surface oxidation, impurities and defects^10,25^. *H*_*c*_ of CoNi microspheres is 107.0 Oe. The *M*_*s*_ and *H*_*c*_ of CoNi@TiO_2_ are 79.6 emu/g and 111.0 Oe, respectively, which are slightly lower than those of CoNi microspheres. The decline of *M*_*s*_ is mainly attributable to the presence of nonmagnetic TiO_2_^11^. After annealed at 600 °C, *M*_*s*_ increases to 94.3 emu/g (Fig. 5b), owing to the elimination of crystal defects and improvement of crystallinity. The increase in *M*_*s*_ is beneficial to the improvement of permeability.

**Figure 6:**
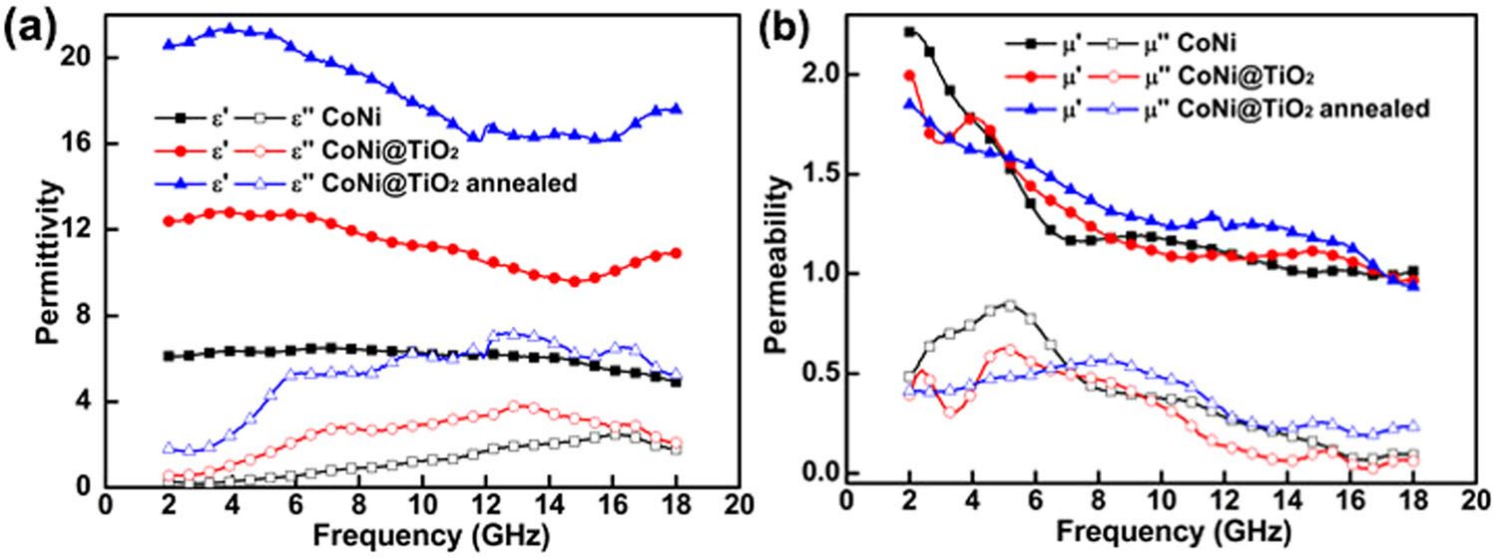
The frequency dependence of (**a**) permittivity and (**b**) permeability for CoNi and CoNi@TiO_2_ microspheres.

The EMA properties of coating are highly dependent on its EM parameters. Fig. 6 shows the frequency dependences of permittivity (*ε*) and permeability (*μ*) of specimens containing CoNi and CoNi@TiO_2_ as fillers. As for CoNi-based sample (Fig. 6a), ε′ does not decline apparently as the frequency increase, while *ε″* increases gradually from 0.3 to 2.5 in 2–16 GHz, before decreases to 1.8 at 18 GHz, revealing mild dielectric relaxation in 9–16 GHz band. Compared with CoNi-based specimen, the *ε′* and *ε″* of specimen containing CoNi@TiO_2_ as fillers are obviously higher all through the frequency range. For instance, *ε′* increases from 6.4 to 12.7, and *ε″* increases from 0.6 to 2.2 at 6 GHz, as shown in Fig. 6a. Meanwhile, the relaxation becomes intense after TiO_2_ coating. After annealed at 600 °C, the permittivity of CoNi@TiO_2_ further increased. For example, *ε′* increases from 12.4 to 20.6, and *ε″* increases from 0.6 to 1.8 at 2 GHz. Moreover, the dielectric relaxation enhances apparently and shifts to 2–12 GHz. As is well known, the permittivity refers to materials’ polarizability, which mainly derives from the interface and dipolar polarizability at microwave frequency^44^. In this case, the evident increase in permittivity is attributed to the enhanced interfacial polarization and the developed dipole polarization. The interfacial polarization arises from the migration of charge carriers on conducting/insulating interfaces according to the Maxwell-Wagner-Sillars theory^45,46^. In this work, CoNi particles dispersed in the paraffin matrix work as charge centers, which can conduce to permittivity on account of interfacial polarization. The coating of TiO_2_ on CoNi microspheres introduces metal/dielectric interfaces and increases the interfacial amount, which would improve interfacial polarization and then promote the permittivity, ultimately, enhance the dielectric loss^47^. During TiO_2_ coating process, the microspheres aggregated together to generate conglomerates of a slender shape. These elongated conglomerates can be considered as a system of dipoles which can induce intense dipole polarization, leading to the enhancement of permittivity^48^. Additionally, the conductivity of CoNi@TiO_2_ microspheres can increase greatly as the defects in the particles eliminates and the crystalline integrity improves during annealing^49^. The improved conductivity is helpful to enhance dielectric relaxation and dipole polarization, leading to the evidently increased permittivity of annealed samples. The enhanced permittivity is believed to be beneficial for the improvement of the dielectric loss and electromagnetic absorption performance^27,50^. Furthermore, the enhanced conductivity could cause conductive loss^51^, which is also beneficial to improve the electromagnetic wave absorption performance of CoNi@TiO_2_ microspheres.

The electromagnetic parameters of CoNi@SiO_2_ were also measured for comparison. Fig. S10a shows the *ε′* and *ε″* as a function of frequency for CoNi@SiO_2_ microspheres in the range of 2–18 GHz. *ε′* together with *ε″* increase obviously in the whole frequency range after SiO_2_ coating, similar to that observed in case of TiO_2_ coating. However, the permittivity of specimen drops evidently after the filled CoNi@SiO_2_ is annealed, which is quite different from that in CoNi@TiO_2_ microspheres. Compared with the specimens containing CoNi@TiO_2_ microspheres as fillers, both *ε′* and *ε″* of CoNi@SiO_2_ are much lower especially when the annealed fillers are applied. For instance, *ε′* and *ε″* are 17.7 and 6.3 for specimens containing annealed CoNi@TiO_2_, 6.0 and 0.7 for specimens containing annealed CoNi@SiO_2_ in 10 GHz, respectively. The interface areas and conductivity dominate dielectric relaxation frequency and intensity, and then administrate the permittivity. The significantly decreased permittivity of CoNi@SiO_2_ annealed microspheres can be ascribed to the reduced interface areas. The well-dispersed CoNi@SiO_2_ microspheres tend to merge together to form large agglomeration, while its spherical shape was maintained. Accordingly, some conductor/insulator interfaces that forms between CoNi cores and SiO_2_ shells disappear, which is supposed to decrease over-all conductor/insulator interface areas. On the other hand, the penetration of H_2_ through SiO_2_ shell to CoNi cores should be difficult as compared with TiO_2_ shell (see TG data in Fig. S11 and associated discussion in Supporting Information), which then blocks the reduction of oxide or the elimination of defects. The improvement of conductivity can thus be limited during annealing, which is quite different from that in case of TiO_2_ coating. The reduced interfaces area together with the restricted conductivity contributes to the decrease in the permittivity. The difference in microstructure evolution, either in the configuration or in the imperfect density, is responsible for the difference in the evolution of EM properties. Consequently, it can be inferred that TiO_2_ coating would endow composite microspheres with better dielectric loss than SiO_2_ coating.

The *μ′* of specimens containing CoNi microspheres as fillers presents an evident decrease from 2 to 7 GHz, and then slight fluctuation in the frequency range of 7–18 GHz, as illustrated in Fig. 6b. The *μ″* exhibits a resonance peak at 5.1 GHz. This characteristic in permeability suggested the natural resonance of CoNi microspheres in the band^52–54^. Besides, the effects from eddy current can be hardly observed all through the band. Particles synthesized via solution chemical method usually have high resistivity, thus the eddy effect can be effectively suppressed. Therefore, the natural resonance is the main magnetic loss mechanism for CoNi microspheres. After TiO_2_ coating, the permeability of CoNi@TiO_2_ decreases slightly. The permeability of ferromagnetic particles basically depends on the *M*_*s*_, thus the slight decrease in *μ* is ascribed to the reduction of *M*_*s*_. Additionally, a distinct broad peak on *μ″* curve at 15–16 GHz for CoNi@TiO_2_ can be observed, which may be associated with the exchange resonance^55^. CoNi particles within local aggregations stacks very densely as intervals below 10 nm, which can be contributed to the exchange resonance. The permeability changes significantly after the fillers annealed, which can be distinguished from the plot shown in Fig. 6b. The *μ′* of annealed fillers increases in most frequency range, which is ascribed to the enhancement of *M*_*s*_. The nature resonance frequency shifts to high frequency range of 8.4 GHz as identified from the *μ″* curve, which would be significant to improve its EMA properties in the microwave range^56^. The presence of SiO_2_ shell did not significantly influence permeability except a slight decrease, which can be distinguished from the plot shown in Fig. S10b.

From the above observations, it can suppose that the incorporation of dielectric TiO_2_ and magnetic CoNi into the electromagnetic wave absorption system had generated massive dielectric and magnetic interactions at materials interfaces, which has a positive impact on the matching of permeability and permittivity^57^. Moreover, the effective complementarity between magnetic loss contributed by CoNi cores and dielectric loss from TiO_2_ shells plays a vital role in the enhancement of electromagnetic wave absorption capability^58^. Therefore, it is possible to enhance the microwave absorption performance of core-shell structure microspheres.

**Figure 7:**
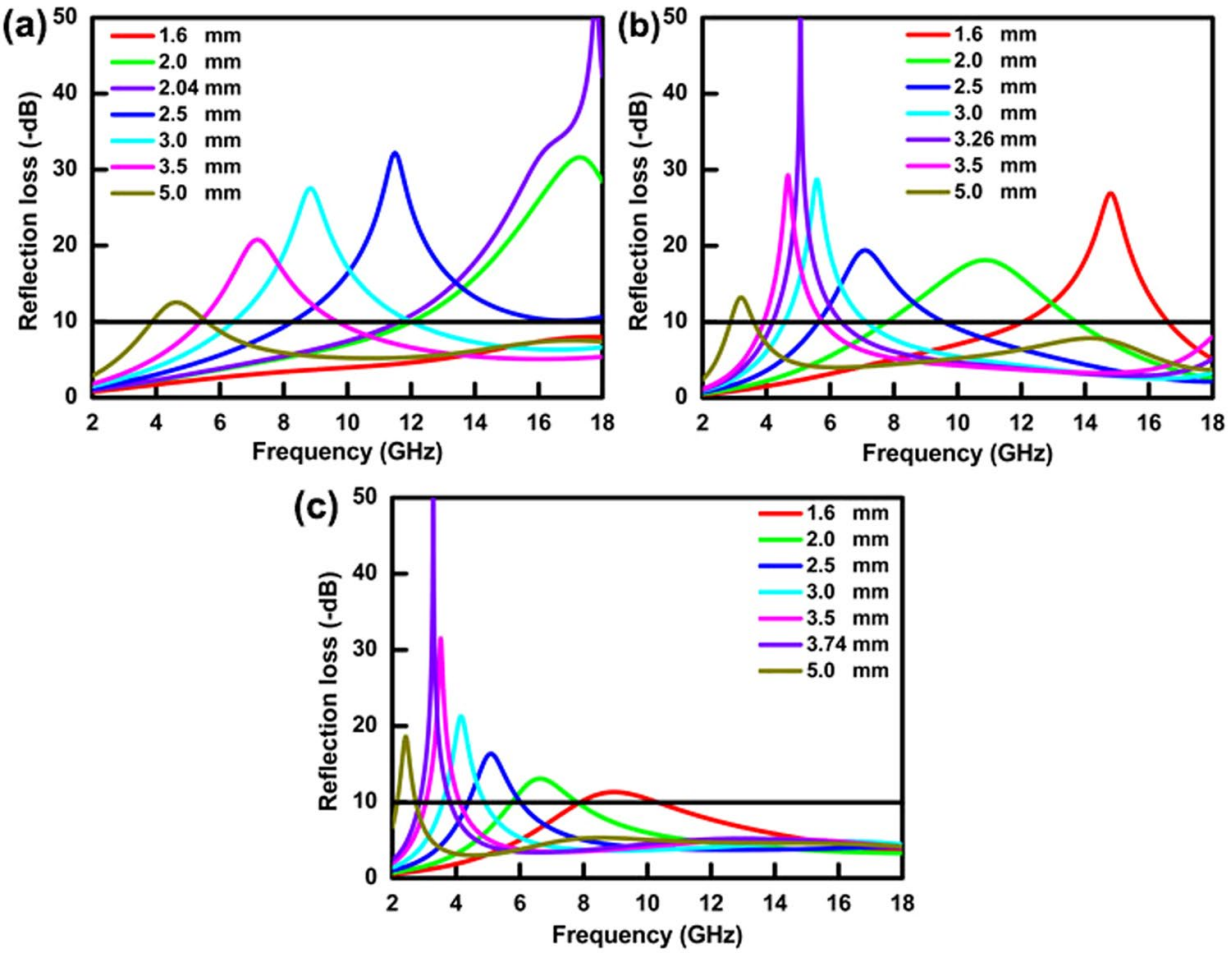
The frequency dependence of reflection loss of CoNi/paraffin composites. (**a**) CoNi microspheres; (**b**) CoNi@TiO_2_ microspheres; (**c**) annealed CoNi@TiO_2_ microspheres.

The reflection loss (*RL*) of CoNi and CoNi@TiO_2_ annealed microspheres are obtained according to the transmit line theory^39,59^. The results are shown in Fig. 7. It can be seen that the microspheres exhibit outstanding microwave absorption performance in terms of a thin absorber layer with a wide frequency bandwidth and strong reflection loss. As shown in Fig. 7a, the maximum *RL* (*RL*_*max*_) for coatings containing CoNi microspheres as fillers is 54.4 dB at 17.8 GHz with a matching thickness of 2.04 mm. Meanwhile, the absorption bandwidth with *RL* higher than 10 dB (*EAB*_10_) is 6.2 GHz (11.8–18 GHz), covering the whole K_u_ band, which is technically significant for the application in K_u_ band. Moreover, an *EAB*_10_ of 9.6 GHz (8.4–18.0 GHz) is observed when a slightly increased matching thickness of 2.5 mm is applied, nearly covering the whole X-K_u_ (8–18 GHz) band. It can be supposed that the excellent microwave absorbing properties of CoNi microspheres is due to its novel conical bulges structure. The surface architecture is an important factor that can tune the microwave absorption capability. The conical bulges on the CoNi microsphere surfaces should have great impacts on the electromagnetic wave absorption performance. The incident electromagnetic wave might suffer multiple scattering in the space among the conical bulges, leading to more intense exhaustion and absorption. Additionally, the large exposed conical bulges would cause strong interfacial magnetic dipole polarization^60^, which may further improve electromagnetic absorption.

The CoNi@TiO_2_ composite microspheres display high EMA properties referring to both the maximum *RL* and the absorption frequency band, as shown in Fig. 7b. *RL*_*max*_ of 59.2 dB was obtained at 5.07 GHz in a coating of 3.26 mm. *RL* higher than 5 dB is 5.8 GHz (3.5–9.3 GHz), covering the whole C band (4–8 GHz). Specifically, *RL* higher than 5 dB of 9.1 GHz is achieved in 4.5–13.6 GHz band when a matching thickness of 2.5 mm is applied. Meanwhile, coating with thickness of 1.6 mm presents *RL* higher than 5 dB of 10.5 GHz in 7.5–18.0 GHz band, covering C, X and K_u_ band, or an *EAB*_10_ of 4.6 GHz in 12.0–16.6 GHz band. It can be seen that the absorption band would shift to much lower frequency if annealed fillers are used, as shown in Fig. 7c. *RL*_*max*_ of 76.6 dB at 3.3 GHz with a thickness of 3.74 mm is obtained, and an absorption bandwidth (*RL* > 5 dB) is 2.3 GHz (2.4–4.6 GHz), nearly covering the whole S band (2–4 GHz). These results indicate that excellent EMA performances can be obtained in S band. Moreover, the absorption bandwidth with *RL* higher than 5 dB is 9.1 GHz in 6.0–15.1 GHz with a thickness of 1.6 mm.

Compared with CoNi@TiO_2_ coating, excellent EMA performance also can be obtained using CoNi@SiO_2_ as fillers. The *RL*_*max*_ is 65.6 dB in 9.2 GHz, and an *EAB*_10_ is 5.5 GHz (6.7–12.2 GHz) with a thickness of 2.75 mm, as described in Fig. S12a. However, the microwave absorption capability slightly declines both in reflection loss and in effective absorption bandwidth of CoNi@SiO_2_ annealed fillers (Fig. S12b). Meanwhile, the absorption band shifts to higher frequency. As described in Fig. S12b, *RL*_*max*_ is 73.8 dB at 17.7 GHz and the *EAB*_10_ is 3.3 GHz from 14.7 to 18.0 GHz with a thickness of 1.82 mm. When the thickness is 1.6 mm, the absorption bandwidth (*RL* higher than 5 dB) is 4.6 GHz (13.4–18.0 GHz), which is much narrower than that of CoNi@TiO_2_ annealed microspheres. From the *RL*_*max*_ curves in Fig. S12c, it can be found that the absorption peaks shift obviously after the introduction of TiO_2_ shells. Upon TiO_2_ coating, microwave absorption moves to S band, indicating excellent EMA performances in these bands. Nevertheless, microwave absorption remains in K_u_ band after SiO_2_ coating. All results indicate that coating of TiO_2_ broadens absorption bandwidth and obtains selective-frequency absorption, demonstrating that construction of core-shell structure is an efficient strategy to improve EMA and tailor strong absorption bands. Table S1 shows the typical CoNi-based composites and their corresponding microwave absorption performances in recent literatures. According to the comparison, the composite microspheres in our study are more competitive than other microwave absorbers for EMA applications in terms of thin thickness and wide frequency range.

## Conclusions

In summary, CoNi microspheres with conical bulges were successfully synthesized via a simple liquid-phase reduction method. CoNi@TiO_2_ core-shell microspheres with prominently enhanced microwave absorption performance were constructed via sol-gel process. Compared with bare CoNi and annealed CoNi@SiO_2_, annealed CoNi@TiO_2_ microspheres display superior microwave absorption performance with *RL*_*max*_ up to 76.6 dB, and the absorption bandwidth of 1.2 GHz in S band. Additionally, the absorption bandwidth (*RL* > 5 dB) can be broaden to 9.1 GHz with a thin thickness of 1.6 mm. The superior EMA properties of CoNi@TiO_2_ core-shell microspheres derive from the intense dielectric loss and magnetic loss. The TiO_2_ shells together with the annealing on one hand ensure CoNi microspheres effective isolation, on the other hand, induce enhanced interfacial polarization and strong dipole polarization to improve the dielectric loss. CoNi@TiO_2_ microspheres demonstrate their excellence on account of the combination of strong magnetic loss from CoNi cores and excellent dielectric loss from TiO_2_ shells. These results ensure that the microspheres in our study with merits of strong absorption and broad effective absorption bandwidths are greatly superior to other CoNi-based EMA fillers. Thus, it is believed that the composites can be used as a promising candidate for high-performance microwave absorbers.

## Methods

All chemicals were of analytical grade and used directly without any pre-treatment. Nickel chloride hexahydrate (NiCl_2_·6H_2_O), cobalt chloride hexahydrate (CoCl_2_·6H_2_O), ethylene glycol (EG), sodium hydroxide (NaOH), hydrazine hydrate (N_2_H_4_·H_2_O, 85%), ammonium hydroxide solution (28 wt%), tetraethyl orthosilicate (TEOS), tetrabutyl orthotitanate (TBOT), acetonitrile and ethanol were all purchased from Sinopharm Chemical Reagent Company.

### Preparation of CoNi microspheres

CoNi spheres were synthesized by a liquid phase reduction process. Typically, 0.01 mol of NiCl_2_·6H_2_O and 0.01 mol of CoCl_2_·6H_2_O were dissolved in 200 mL of EG under mechanical stirring at 85 °C, followed by the addition of 0.12 mol of NaOH. After 20 min, 8 mL of N_2_H_4_·H_2_O was added. The reaction duration is 1 h. The obtained products were washed for several times with distilled water and absolute ethanol. Finally, the products were dried in a vacuum oven at 60 °C overnight for further characterization.

### Preparation of CoNi@TiO_2_ microspheres

0.5 g of as-prepared CoNi microspheres were dispersed in the mixture solvent containing ethanol (180 mL) and acetonitrile (60 mL). The mixture was ultrasonicated for 30 min, followed by the addition of 1 mL of ammonia aqueous solution under mechanical stirring. Afterward, 0.5 mL of TBOT was added, and the reaction was allowed to proceed for another 2 h. The black particles were collected and washed with ethanol, and then dried at 60 °C.

### Preparation of CoNi@SiO_2_ microspheres

0.5 g of CoNi microspheres were dispersed in ethanol (160 mL) and deionized water (40 mL), and sonicated for 30 min. Then, 4 mL of ammonia aqueous solution was added under mechanical stirring. Afterward, 0.2 mL of TEOS was added, and the reaction was allowed to occur for 4 h. The resulted precipitates were collected and washed with absolute ethanol, and dried at 60 °C. The as-prepared CoNi@TiO_2_ and CoNi@SiO_2_ microspheres were annealed at 600 °C for 2 h under H_2_ atmosphere for microstructure tailoring.

### Characterization

The crystal structure of as-prepared products was characterized by X-ray diffraction (XRD, Rigaku D/max-rB, Cu K_*α*_). The morphologies of microspheres were characterized using a field-emission scanning electron microscope (SEM, FEI Quanta 200 F) equipped with an energy dispersive spectrometer (EDS), and a transmission electron microscope (TEM, JEOL JEM-2100). The element values in the samples were analyzed on X-ray photoelectron spectroscopy (XPS, Thermo Fisher Scientific VG K_α_ Probe) using Al K_α_ radiation as the excitation source. The magnetic properties of the powder samples were measured by a vibrating sample magnetometer (VSM, Lakeshore 7300) at room temperature. The permittivity and permeability of samples in 2–18 GHz range were examined with a vector network analyzer (VNA, Agilent N5230A). For testing, 70 wt.% CoNi particles were homogeneously dispersed in paraffin matrix. Thermogravimetry curves of composite microspheres were recorded on a thermal gravimetric analyzer (TG, SDT Q600 V20.9 Build 20) under air from room temperature to 800 °C with a ramping rate of 10 °C min^−1^.

## Electronic supplementary material

Supporting Information
