# Supplementary material for: Rational Construction of Uniform CoNi-Based Core-Shell Microspheres with Tunable Electromagnetic Wave Absorption Properties
Source: Sci Rep. 2018 Feb 16;8:3196. doi: 10.1038/s41598-018-21047-z (PMC5816601; doi:10.1038/s41598-018-21047-z)
Supplement: Supplementary file 1 — Supporting Information [file 41598_2018_21047_MOESM1_ESM.doc]

*Supporting Information for*

Rational Construction of Uniform CoNi-Based Core-Shell Microspheres with Tunable Electromagnetic Wave Absorption Properties

Na Chen,^1,2^ Jian-Tang Jiang,^1^ Cheng-Yan Xu,^1,2^ Shao-Jiu Yan,^3^ and Liang Zhen^1,2^

^1^School of Materials Science and Engineering, Harbin Institute of Technology, Harbin, 150001, China, ^2^MOE Key Laboratory of Micro-System and Micro-Structures Manufacturing, Harbin Institute of Technology, Harbin 150080, China, ^3^Beijing Institute of Aeronautical Materials, Beijing 100095, China

Correspondence and requests for materials should be addressed to J.-T.J. ([jjtcy@hit.edu.cn](mailto:jjtcy@hit.edu.cn)) or L.Z. (lzhen@hit.edu.cn)


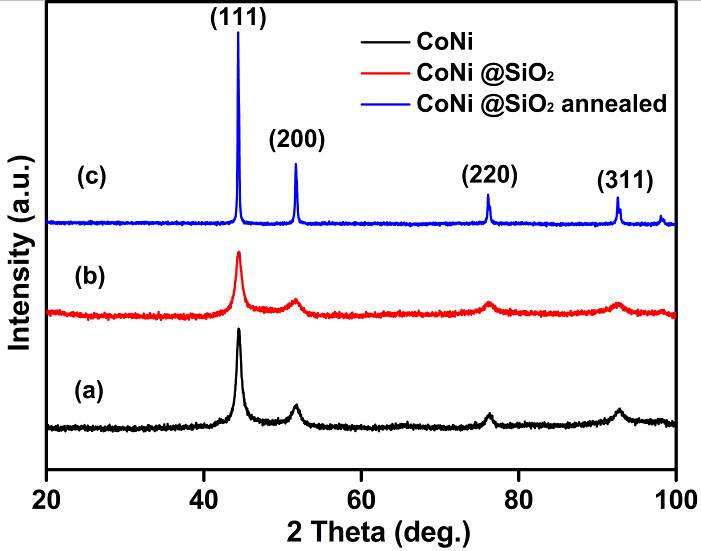


**Figure S1.** XRD patterns of (a) CoNi, (b) CoNi@SiO_2_ and (c) annealed CoNi@SiO_2_ microspheres.


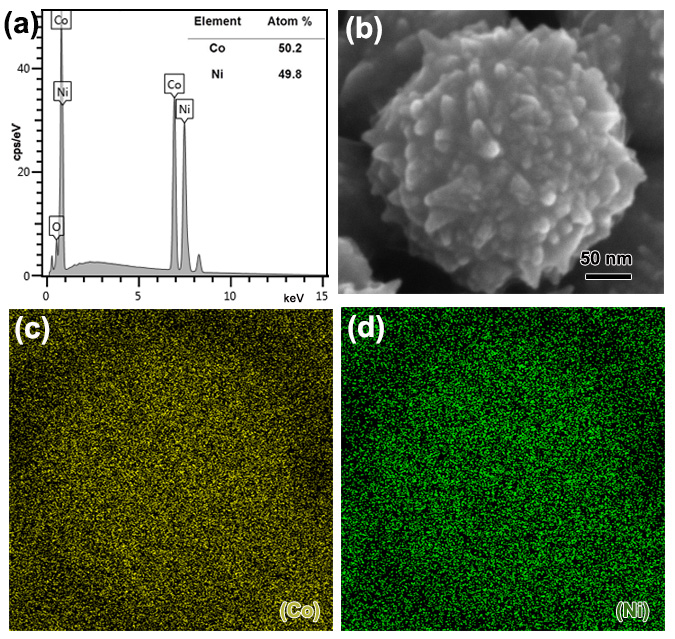


**Figure S2.** (a) EDS spectrum, (b) SEM image of an individual CoNi microsphere, and element mappings of (c) Co, (d) Ni.


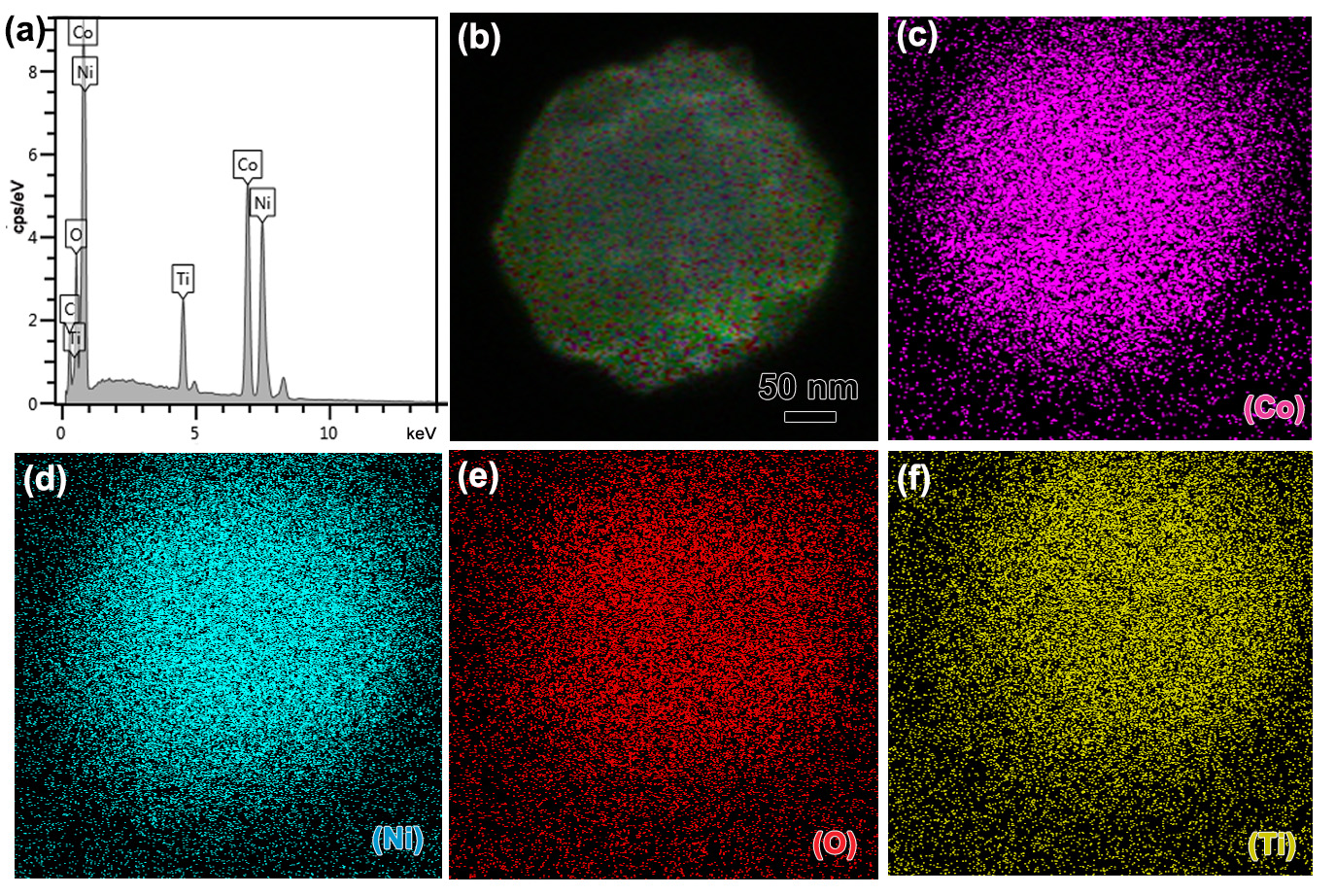


**Figure S3.** (a) EDS spectrum, (b) SEM image of an individual CoNi@TiO_2_ microsphere, and element mappings of (c) Co, (d) Ni, (e) O and (f) Ti.


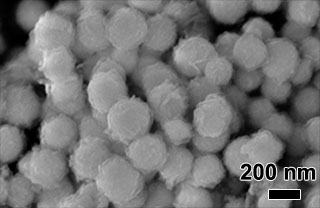


**Figure S4.** SEM image of CoNi@TiO_2_ microspheres annealed at 600 °C.


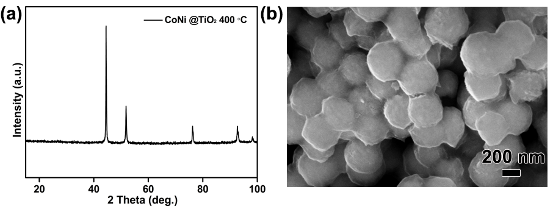


**Figure S5.** (a) XRD pattern and (b) SEM image of CoNi@TiO_2_ microspheres annealed at 400 °C.

CoNi@TiO_2_ microspheres were annealed at 400 °C and characterized with XRD and SEM analysis, as shown in Figure S5. XRD peaks corresponding to crystalline TiO_2_ cannot be observed in the XRD pattern, indicating that TiO_2_ shells retain the amorphous structure when annealed at low temperature (Figure S5a). SEM image in Figure S5b shows that the morphology of CoNi@TiO_2_ microspheres was well retained after annealed at 400 °C.


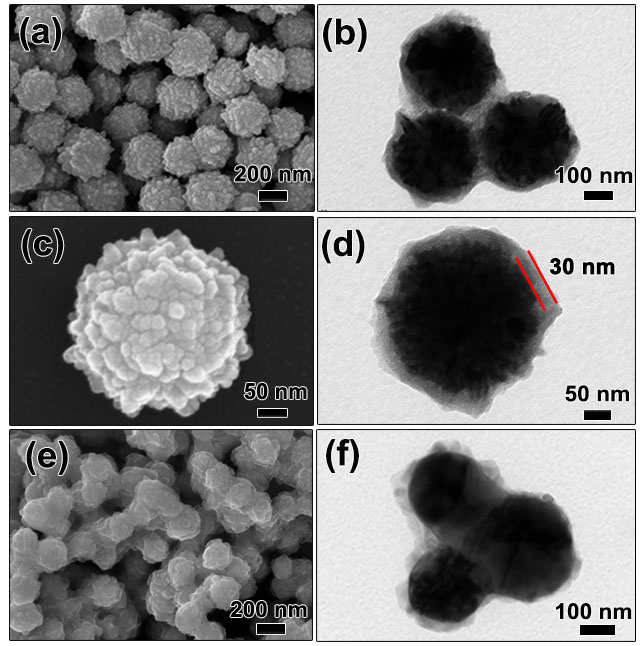


**Figure S6.** (a, c) SEM and (b, d) TEM images of CoNi@SiO_2_ microspheres. (e) SEM and (f) TEM images of CoNi@SiO_2_ annealed at 600 °C.


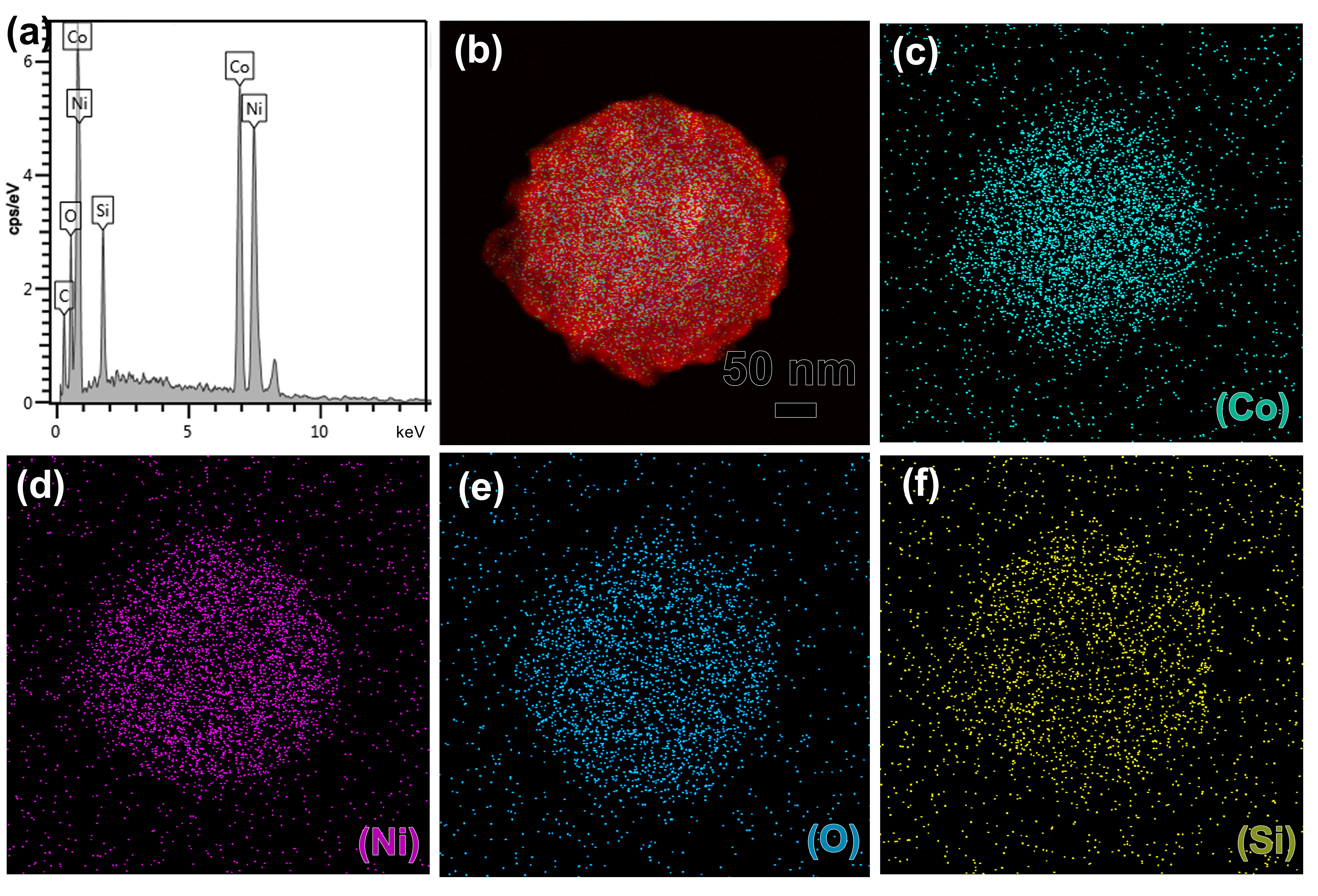


**Figure S7.** (a) EDS spectrum, (b) SEM image of an individual CoNi@SiO_2_ microsphere, and element mappings of (c) Co, (d) Ni, (e) O and (f) Si.


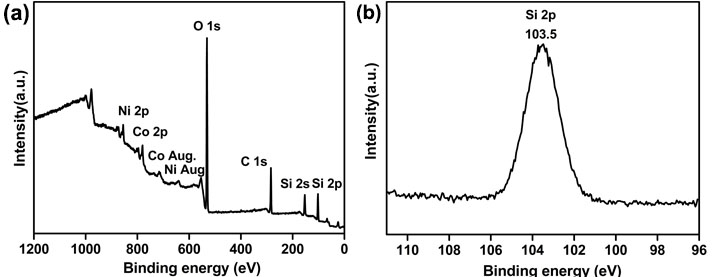


**Figure S8.** (a) XPS survey spectrum of CoNi@SiO_2_ microspheres. (b) Si 2p spectrum of CoNi@SiO_2_.


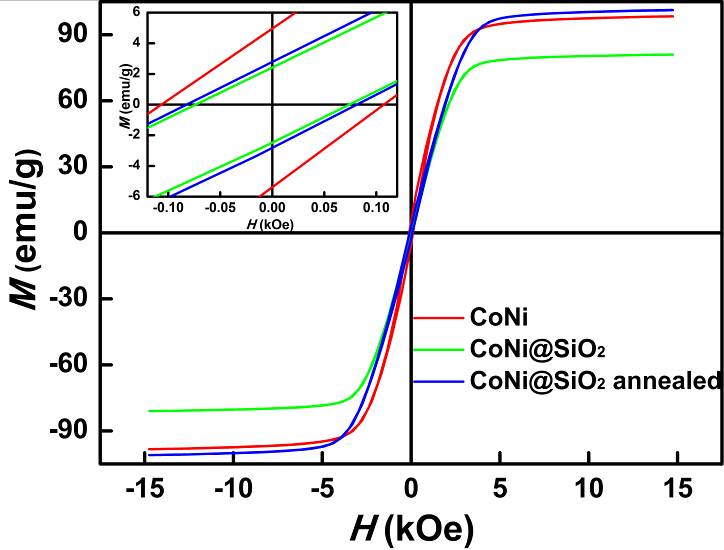


**Figure S9.** Hysteresis loops of CoNi, CoNi@SiO_2_ and CoNi@SiO_2_ annealed microspheres measured at room temperature. The *M_s_* and *H_c_* are 81.0 emu/g and 73.8 Oe, respectively. After annealed at 600 °C, *M_s_* increases to 101.2 emu/g.


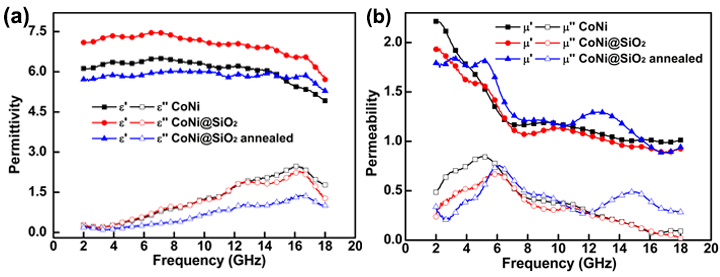


**Figure S10.** The frequency dependence of (a) permittivity and (b) permeability for CoNi and CoNi@SiO_2_ annealed microspheres.


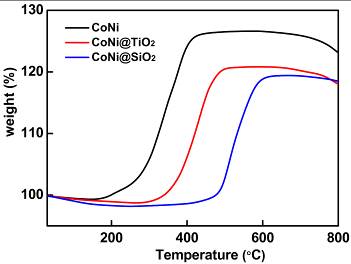


**Figure** **S11** TG patterns of as-prepared CoNi microspheres, CoNi@TiO_2_ and CoNi@SiO_2_ core-shell microspheres.

TG curves in Figure S11 reveal that the mass of CoNi microspheres increases apparently after the exposure temperature was increased to beyond 170 °C. Upon TiO_2_ coating, CoNi microspheres are protected, and the mass of CoNi@TiO_2_ is increased to about 300 °C. Compared with TiO_2_ coating, the exposure temperature is increased to 480 °C for CoNi@SiO_2_ microspheres, suggesting that SiO_2_ shell should effectively prevent the penetration of air to CoNi particles. Accordingly, the penetration of H_2_ into CoNi cores of CoNi@TiO_2_ microspheres was supposed to be much easier during the hydrogen-thermal annealing as compared with CoNi@SiO_2_ microspheres, which can help to eliminate the non-conducting inclusion and lattic defects.


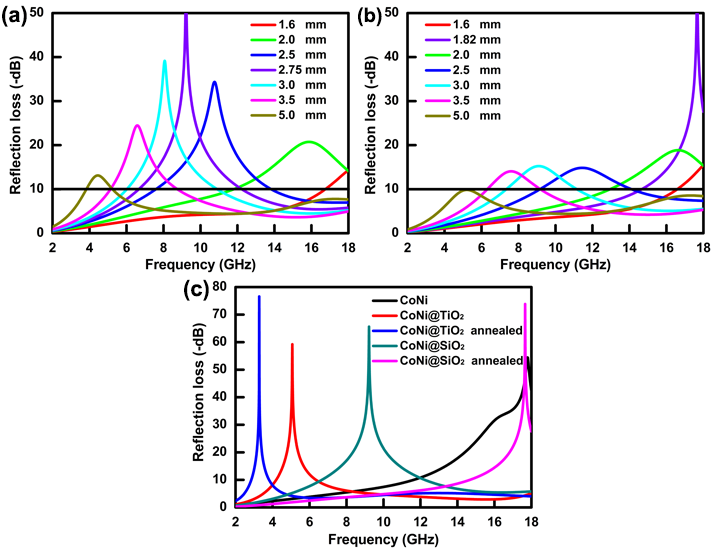


**Figure S12.** The frequency dependence of reflection loss of (a) CoNi@SiO_2_/paraffin composites and (b) annealed CoNi@SiO_2_/paraffin composites. (c) The comparison of maximum RL of the composites.

**Table S1 Typical CoNi-based composites for electromagnetic absorption in recent literature:**

| Sample | *RL_max_* (dB) | Optimum thickness (mm) | Optimum  frequency (GHz) | Frequency range  (RL < -10 dB) | Ref. |
| --- | --- | --- | --- | --- | --- |
| urchin-like CoNi microspheres | 33.5 | 5.0 | 3.0 | 2.3-4.0 (1.7) | S1 |
| flower-like CoNi microspheres | 21.8 | 2.0 | 8.0 | 6.0-11.0 (5.0) | S1 |
| chain-like CoNi | 34.33 | 1.0 | 17.5 | 15.2-18.0 (2.8) | S2 |
| CoNi microflowers | 28.5 | 2.0 | 6.8 | 5.0-10.5 (5.5) | S3 |
| CoNi@C nanocapsules | 35 | 2.0 | 16.2 | 12–18 (6.0) | S4 |
| Co_20_Ni_80_@TiO_2_ microspheres | 25 |  | 5.9 | 10–16.2 (6.2) | S5 |
| CoNi/NG hybrids | 22 | 2.0 | 10.0 |  | S6 |
| CoNi/rGO composites | 23.3 | 6.0 | 17.5 | 16.0-18.0 (2.0) | S7 |
| CoNi | 54.4 | 2.04 | 17.8 | 12.2–18.0 (5.8) | this work |
| CoNi@SiO_2_ annealed | 73.8 | 1.82 | 17.7 | 14.7-18.0 (3.3) | this work |
| CoNi@TiO_2_ annealed | 76.6 | 3.74 | 3.3 | 2.7-3.9 (1.2) | this work |

**References**

1. Liu, Q. *et al.* Dependency of magnetic microwave absorption on surface architecture of Co_20_Ni_80_ hierarchical structures studied by electron holography. *Nanoscale* **7**, 1736-1743 (2015).
2. Zhao, B., Shao, G., Fan, B., Xie, Y. & Zhang, R. Preparation and electromagnetic wave absorption of chain-like CoNi by a hydrothermal route. *J. Magn. Magn. Mater.* **372**, 195-200 (2014).
3. Liu, Q. *et al.* Insights into size-dominant magnetic microwave absorption properties of CoNi microflowers via off-axis electron holography. *ACS Appl. Mater. Interfaces* **7**, 4233-4240 (2015).
4. Wang, H. *et al.* Broadband microwave absorption of CoNi@C nanocapsules enhanced by dual dielectric relaxation and multiple magnetic resonances. *Appl. Phys. Lett.* **102**, 223113 (2013).
5. Chen, C. *et al.* Fabrication of hierarchical TiO_2_ coated Co_20_Ni_80_ particles with tunable core sizes as high-performance wide-band microwave absorbers. *Phys. Chem. Chem. Phys.* **18**, 26712-26718 (2016).
6. Feng, J. *et al.* Interfacial interactions and synergistic effect of CoNi nanocrystals and nitrogen-doped graphene in a composite microwave absorber. *Carbon* **104**, 214-225 (2016).
7. Guo, X. Q., Bai, Z. Y., Zhao, B., Zhang, R. & Chen, J. B. Microwave absorption properties of CoNi nanoparticles anchored on the reduced grapheme oxide. *J. Mater. Sci.: Mater. Electron.* **27**, 8408-8415 (2016).
